# Aqueous extract of *Sapindus mukorossi* induced cell death of A549 cells and exhibited antitumor property *in vivo*

**DOI:** 10.1038/s41598-018-23096-w

**Published:** 2018-03-19

**Authors:** Min Liu, Yen-Lin Chen, Yao-Haur Kuo, Mei-Kuang Lu, Chia-Ching Liao

**Affiliations:** 1 Department of Life Science, Chinese Culture University, Taipei, Republic of China; 2 Graduate Institute of Biotechnology, Chinese Culture University, Taipei, Republic of China; 3 Ministry of Health and Welfare, National Research Institute of Chinese Medicine, Taipei, Republic of China; 4 Graduate Institute of Integrated Medicine, China Medial University, Taichung, Republic of China; 5 Department of Horticulture and Biotechnology, College of Agriculture, Chinese Culture University, Taipei, Republic of China; 6 Department of Biology, Chinese Culture University, Taipei, Republic of China

## Abstract

*Sapindus mukorossi* is a deciduous plant and has recently been recognized to have anticancer property. In the present study, we discovered that *S*. *mukorossi* leaf and stem aqueous extract (SaM) contained two polysaccharides mainly made of myo-inositol, galactose, glucose, and fructose and the aim of this study was to investigate the antitumor property the aqueous extract SaM. *In vitro* treatment of SaM diminished proliferative potential of lung adenocarcinomic cells and induced intracellular oxidative stress, as well as necrotic cell death. Moreover, exposure to SaM attenuated cell migration, demonstrating the effectiveness at reducing invasive property of malignant lung cells. Gene and protein expression studies indicated that SaM treatment altered the expression of proliferation/survival modulator NF-κB, tumor growth modulator ERK2, metastasis-associated molecules MMP9/12, and tumor suppressor p53 in A549 cells. Using model animals bearing Lewis lung cancer cell LL/2, we demonstrated that SaM was antitumoral and did not induce any undesired organ damage, immunotoxicity, and off-target inflammation. This work, to our knowledge, is the first study documents the antitumor bioactivity of aqueous extract riched in polysaccharides from *S*. *mukorossi* and provides insights into the potential pharmacological application of SaM as antitumor agent against lung cancer.

## Introduction

*Sapindus mukorossi* (Sapindaceae), also known as the soapnuts, is a deciduous plant widely distributed in the tropical and sub-tropical regions of Asia. *S*. *mukorossi* is economically valuable, which contains natural surfactants for making commercial ingredient of shampoo and cleaners^1^. The presence of triterpenoid saponins^2–4^, fatty acids^5^, and flavonoids^6^, from the pericarp, stem, and fruit of the plant have been reported. Recently, pharmacological properties of *S*. *mukorossi* are explored to show that the plants possess antimicrobial, cytotoxic, molluscicidal, insecticidal, fungicidal, and spermatocidal activities^7^. Moreover, hepatoprotective, anti- inflammatory, and antitumor properties of *S*. *mukorossi* have been reported. In addition to these pharmacological properties, *S*. *mukorossi* has been demonstrated to be antitumoral against several types of tumor such as liver carcinomic Hepa59T/VGH cells, large lung carcinomic NCI cells, cervical epithelioid carcinomic HeLa cells, medulloblastoma Med/Daoy, colon adenocarcinomic WiDr cells, and oral epidermoid carcinomic KB cells^8,9^.

Lung cancer is considered to be one of the most deleterious human malignancies in the modern time and it is also the leading cause of cancer-related mortalities in both genders, accounting for 15% of all cancer deaths globally. Lung cancer is generally classified into two main types: the non-small cell lung cancer (NSCLC; 80% of diagnosed cases) and the small cell lung cancer (SCLC; 20% of diagnosed cases). Despite all our advances in managing cancers, providing a curative therapy regimen for the patients with non-small lung cancer remains to be a challenge to many oncologists. Patients with NSCLC usually have to undergo intensive surgery treatment depending on the disease stage at diagnosis and the patient’s performance status. Nevertheless, nearly all cases of NSCLC require chemotherapy even if the initial surgery is potentially curative and chemotherapeutic regimen is usually the only disease management option for those at advanced stage. Although the chemotherapy-based treatment has tremendously improved the symptoms and quality of life of patients with NSCLC, the overall survival rate still remains at a low level.

Research focuses on the use of natural products for treating cancers has offered possible alternatives for some patients. Therapeutic agents derived from several herbal plants, such as *Platycodon grandiflorum* (Campanulaceae), *Morus alba* (Moraceae), *Rhus verniciflua* (Anacardiaceae), *Perilla frutescens* (Labiatae), *Stemona japonica* (Stemonaceae), *Tussilago farfara* (Compositae) and *Draba nemorosa* (Brassicaceae), have been used conventionally as folk remedies for treating lung diseases, including cancer^10^.

In this study, we discovered the aqueous extract of *S*. *mukorossi* stem and leaf, named as SaM, is rich in polysaccharides. The purpose of this study was to assess the antitumor property of SaM against diseased lung cells. *In vitro* cytotoxicity of SaM was evaluated in A549 cells with NSCLC origin. *In vivo* toxicological assessment was carried out to evaluate the safety use of SaM and antitumor activity of SaM was examined using Lewis lung carcinoma cells (LL/2) inoculated ICR mice. Our data showed that SaM not only diminished the proliferative potential of A549 cells but also induced intracellular oxidative stress and necrotic cell death. Moreover, exposure to SaM attenuated cell migration and altered the expression of proliferation/survival modulator NF-κB, tumor growth modulator ERK2, metastasis-associated molecules MMP9/12, and tumor suppressor p53 in A549 cells. Using model animals bearing Lewis lung cancer cell LL/2, we demonstrated that SaM was antitumoral and did not induce any undesired organ damage, immunotoxicity, and off-target inflammation.

## Results and Discussion

### SaM composition analysis

**Figure 1:**
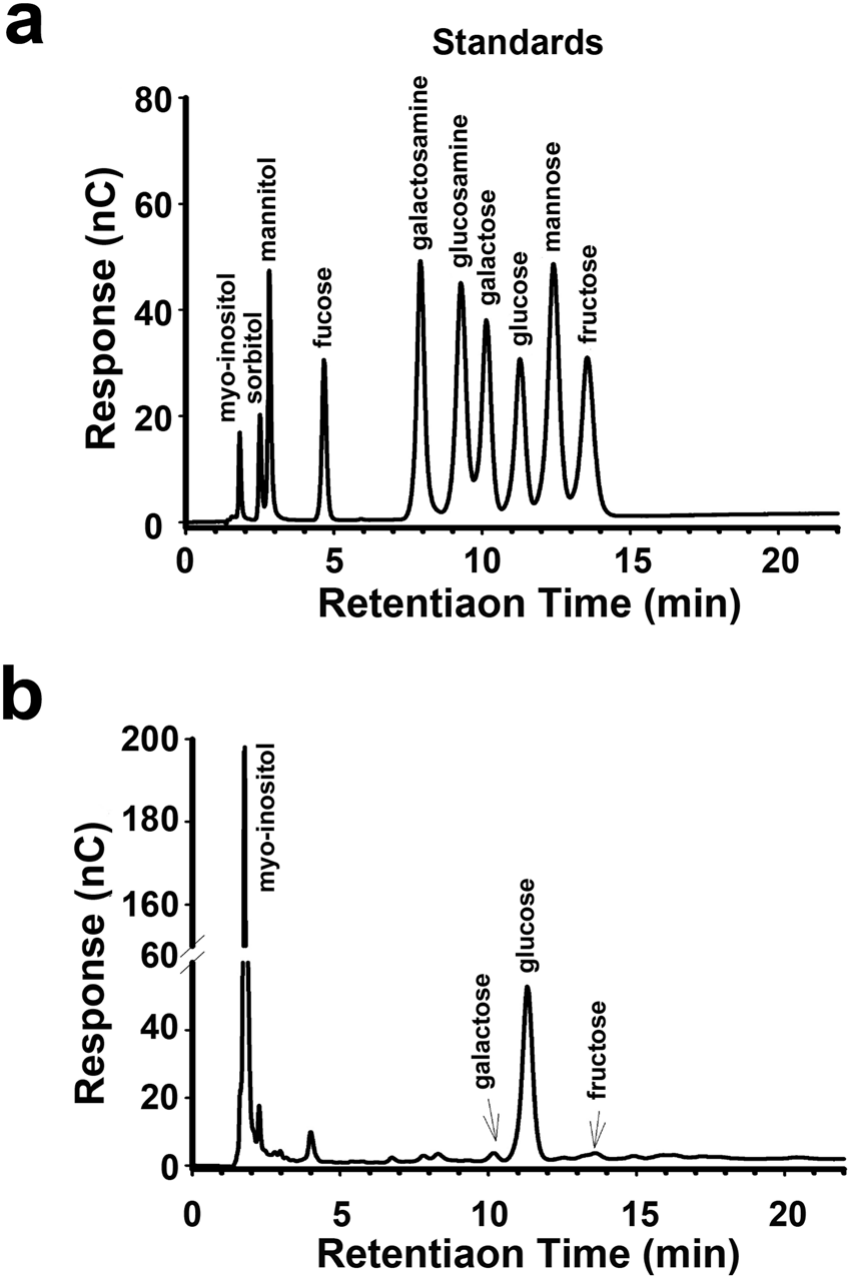
High-performance anion-exchange chromatography (HPAEC) of SaM. (**a**) Monosaccharide standards; (**b**) Chromatogram of SaM. The HPAEC analysis was carried out in 18 mM NaOH for 22 minutes at ambient temperature.

Most analytical studies of *S*. *mukorossi* focus on one of its major component saponin. Interestingly, our H^+^ NMR analysis did not detect saponins in SaM (Supplementary Figure S1A); instead, SaM mainly contained two polysaccharides with molecular weight ranging from >3000 Da (in SaM fraction I) and >89000 Da (in SaM fraction II) (Supplementary Figure S1B). Data from acid hydrolysis of SaM followed by high-performance anion-exchange chromatography (HPAEC) indicated that the polysaccharides were mainly made of myo-inositol and glucose (Fig. 1). As shown in Table 1, the carbohydrate composition of SaM contained myo-inositol, galactose, glucose, and fructose in the value of 127.30, 8.38, 297.98, and 27.64 μmol/g.

**Table 1:** Carbohydrate composition analysis of SaM.

	Neutral sugars (μmol/g)
myo-inositol	127.30 ± 0.01
galactose	8.38 ± 0.06
glucose	297.98 ± 1.18
fructose	27.64 ± 0.03

### *In Vitro* Assessment of Cytotoxic Property

**Figure 2:**
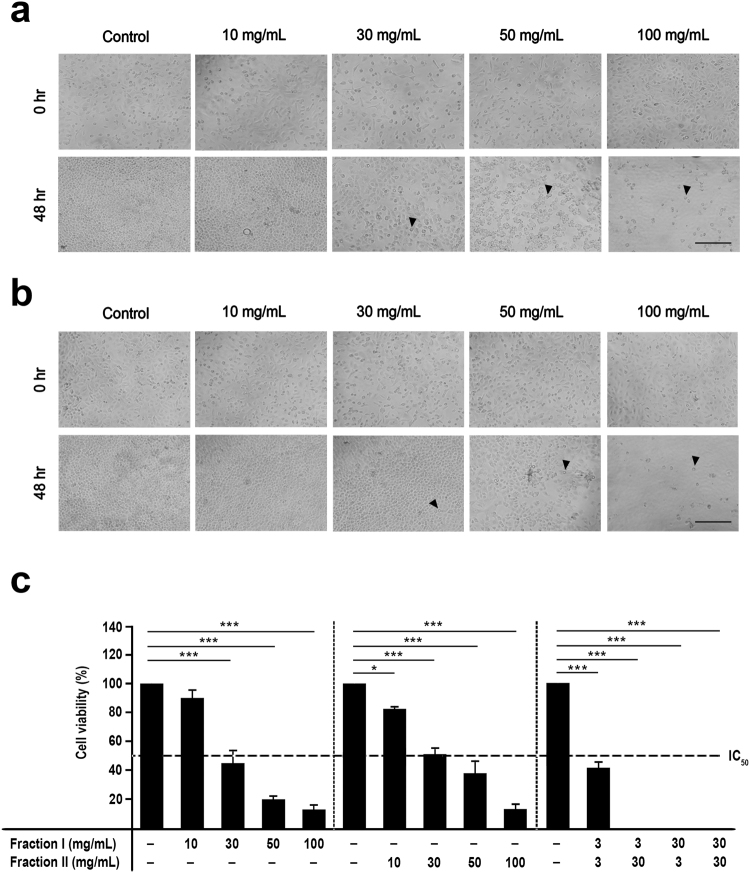
Analysis of cell viability of A549 cells cultured with fraction I and II of SaM. The experimental groups were administered with SaM fraction I and II for 48 hours (10 to 100 mg/mL). Light micrographs of SaM fraction I (**a**) and II (**b**) treated A549 cells on day 3. Cell irregularity in shape were observed (shown with arrowheads). Images were taken at 100× magnifications. (**c**) Cell viability analysis of control and treated cells. Data is expressed as mean percentage ± SEM with respect to untreated cells (control), which is set to 100%. IC_50_ = the half maximal inhibitory concentration. Scale bar = 200 μm. Statistical differences between the control and the treated groups were determined by a one-way ANOVA followed by the Dunnett’s post-hoc test when results of the ANOVA were significant: *p < 0.05 vs. control, ***P < 0.001 vs. control.

**Figure 3:**
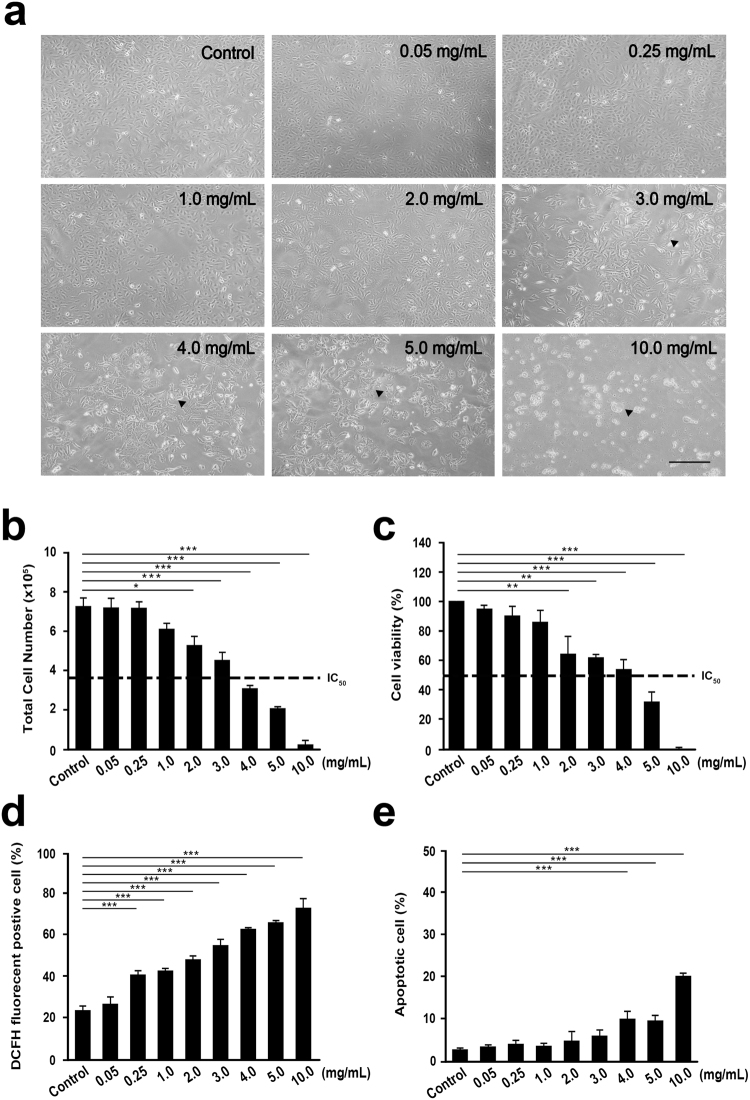
Analysis of cell proliferation, viability, oxidative stress, and apoptosis of A549 cells cultured in the presence or absence of SaM. The experimental groups were administered with SaM (0.05 to 10 mg/mL) for 48 hours. Control groups were not exposed to SaM. (**a**) Light micrographs of control and treated A549 cells on day 3. Cell detachment, cell rounding, and irregularity in shape were observed (shown with arrowheads). Images were taken at 100× magnifications. (**b**) Cell proliferation analysis of control and SaM treated A549 cells. Data are expressed as total number of harvested cells (x10^5^ cells) on day 3. (**c**) Cell viability analysis of control and treated cells. Data is expressed as mean percentage ± SEM with respect to untreated cells (control), which is set to 100%. (**d**) Evaluation of intracellular hydrogen peroxide production (oxidative stress) by DCFH assay. The value represents the average percentage of DCFH fluorescence positive cell ± SEM. (**d**) Cell apoptosis: results are expressed as mean percentage (apoptotic cells/total cells) ± SEM. IC_50_ = the half maximal inhibitory concentration. Scale bar = 100 μm. Statistical differences between the control and the treated groups were determined by a one-way ANOVA followed by the Dunnett’s post-hoc test when results of the ANOVA were significant: *P < 0.05, **P < 0.01, ***P < 0.001 vs. control.

Recent research has demonstrated that carbohydrate-based therapeutic treatment is promising in cancer management^11^. Therefore, it was our great interest to characterize the antiproliferative and cytotoxic property of our aqueous extract SaM, which was rich in polysaccharides. Our cytotoxicity analysis showed that A549 cell morphology were adversely affected by the present of SaM fraction I or II (Fig. 2a,b) and the cell viability was also significantly reduced after 48 hours exposure (Fig. 2c). Fraction I was effective at ≥30 mg/mL whereas fraction II was effect at ≥10 mg/mL, and IC_50_ for both fractions were ~30 mg/mL. Interestingly, we noted that the addition of ≥3 mg/mL SaM (containing both fraction I and II) induced cell swelling, irregularity in cell shape, cell rounding, and eventually cell detachment (Fig. 3a; shown with arrowheads). Cell proliferative potential of A549 cells was significantly compromised when cells were exposed to ≥2 mg/mL SaM in a dose-dependent manner and the IC_50_ value was estimated to be ~3–4 mg/mL (Fig. 3b). As shown in Fig. 3c, treatment with SaM (≥2 mg/mL) showed a significant inhibitory effect on A549 cell viability and the IC_50_ value was estimated to be ~3–4 mg/mL. The IC_50_ of SaM was ~10 times lower than that of SaM fraction I or II alone (Fig. 2c). It is possible that bioactive components, including polysaccharides, from both fractions are required to reduce A549 cell viability. It is also likely that constituents from one of the fractions sensitize A549 cells, allowing cells to become more susceptible to the bioactive compounds of the other fraction. This proposed mechanism of action has been supported by studies of anticancer carbohydrate such as brown seaweed fucoidan and structural polysaccharide hyaluronan. In fact, co-treatment of fraction I and II (Fig. 2c) showed synergistic-manner inhibition of A549 cell viability and CI index calculation confirmed the synergy (CI index of fraction I/II: 3 mg/3 mg = 0.217; 3 mg/30 mg = 0.049; 30 mg/3 mg = 0.089; 30 mg/30 mg = 0.12). For this reason, the cytotoxic property of SaM was further investigated.

Based on the proceeding findings, SaM was cytotoxic and antiproliferative to A549 cells. We postulated that the induction of intracellular oxidative stress, which eventually leads to cell death, was responsible for observed inhibitory effect. Indeed, a significant elevation of intracellular H_2_O_2_ production in treated A549 cells was evident as cells exposed to very low dose (≥0.25 mg/mL) of SaM (Fig. 3d). The SaM concentration (≥0.25 mg/mL) at which effectively induced the onset of H_2_O_2_ production in A549 cells was 8-fold lower than the concentration at which adversely affected the cell proliferation and viability (≥2 mg/mL), suggesting that the induction of oxidative stress is one of the underlying anticancer mechanisms in A549 cells.

**Figure 4:**
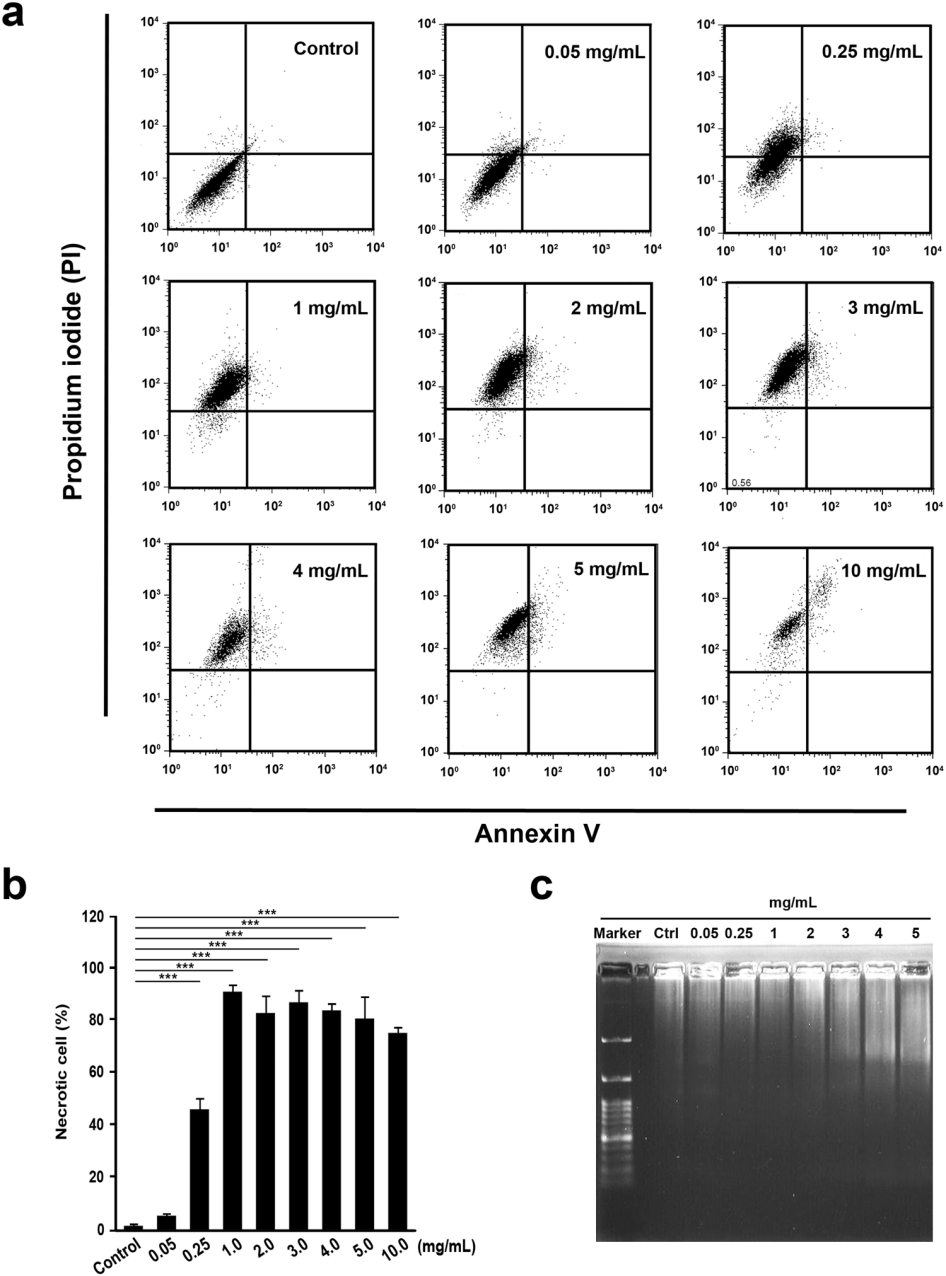
Cell necrosis analysis of A549 cells by Annexin V-PI flow cytometric and DNA fragmentation assays. The experimental groups were administered with SaM (0.05–10 mg/mL) for 48 hours. Control groups were not exposed to SaM. (**a**) The representative flow cytometric patterns of control and treated cells stained with Annexin V and PI are shown. (**b**) Cell necrosis: results are expressed as mean percentage (apoptotic cells/ total cells) ± SEM. (**c**) DNA fragmentation analysis of SaM treated A549 cells. Statistical differences between the control and the treated groups were determined by a one-way ANOVA followed by the Dunnett’s post-hoc test when results of the ANOVA were significant: ***P < 0.001 vs. control.

To delineate the type of cell death that was responsible for SaM cytotoxic property we observed, PI/annexin V analysis was performed. We noted that only a slight but significant increase in apoptosis when A549 cells were treated with high dose (≥4 mg/mL) of SaM (Fig. 3e). However, administration of very low dose of SaM (0.25 mg/mL) induced necrosis in more than 40% of cells and almost 100% of cells were necrotic when exposed to ≥1 mg/mL SaM (Fig. 4a,b). The induction of necrosis cell death was also supported by the observation that DNA of treated A549 cells was fragmented and exhibited smear pattern in electrophoresis assay (Fig. 4c). Prior to 1980s, necrosis had been described as uncontrolled type of cell death due to tissue acute trauma, which eventually led inflammation. Recently studies have redefined this type of cell death and demonstrated that necrosis (also known as necroptosis) can be indeed programmed by the activation of several key modulators^6^. In contrast to necrosis, apoptosis is defined as an orchestrated cell death and is much favored mechanism in eradicating human cancer cells. However, the efficacy of this pro-apoptotic therapy is limited by drug resistance due to disrupted apoptosis machinery, overactive pro-survival signaling pathways, increased expression of therapeutic target, activation of alternative compensatory pathways, a high degree of molecular heterogeneity in tumor cells, upregulation of drug transporters, and multidrug resistance^2,3^. In cancer cells, genetic mutations and abnormal gene expression are common in the extrinsic and intrinsic apoptotic pathways. Since necrotic and apoptotic pathway use different set of components, cancer cells, that are resistant to apoptosis-inducing agents, may be sensitive to necrosis-inducing agents. Therefore, necrosis-based cancer therapy might be a novel alternative way of tackling apoptosis-resistant cancer cells as long as undesired side-effect such as systematic inflammation is not induced.

**Figure 5:**
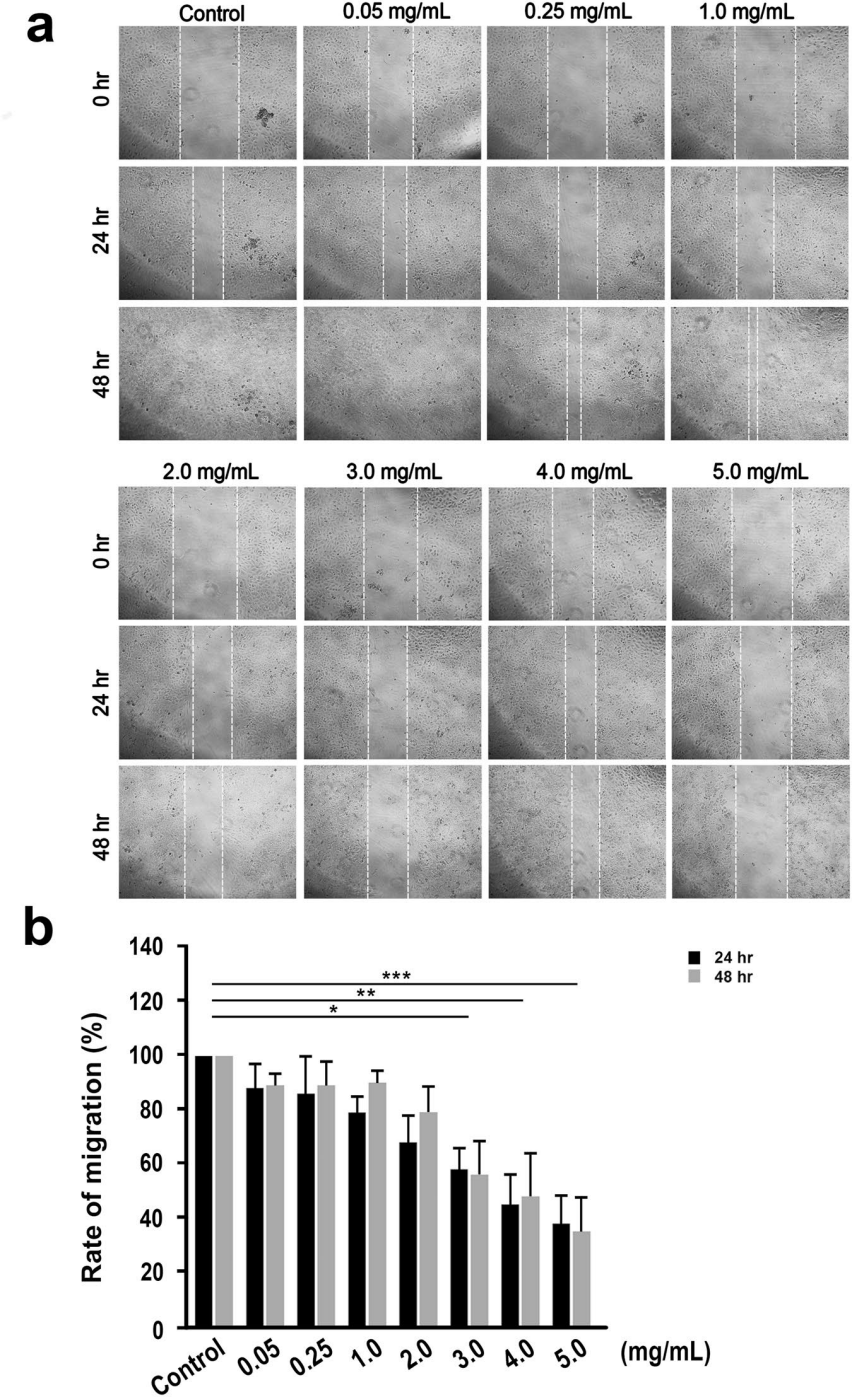
Cell migration of A549 cells treated with SaM. (**a**) Representative images of cell migration by scratch-wound healing assay. (**b**) Rate of migration is expressed as mean percentage (average migrated distance of treated cells/average migrated distance of control cells). Average migration distance was measured at least 20 readings of distance for each sample. Solid bar = 24 h and grey bar = 48 h. Statistical differences between the control and the treated groups were determined by a one-way ANOVA followed by the Dunnett’s post-hoc test when results of the ANOVA were significant: *p < 0.05, ***P < 0.001 vs. control.

Studies have reported adverse and fatal cell dissemination in human adenocarcinomic lung cells to common sites such as the brain, bones, adrenal glands, liver, kidneys, and the other lung. Therefore, one of the key challenges of this study was to test whether SaM attenuates malignant cell migration and reduces the invasiveness of cancer cells. Remarkably, the rate of migration was significantly reduced when A549 cells were treated with ≥3 mg/mL of SaM for 24 and 48 hours in a dose-dependent manner, demonstrating the effectiveness of SaM in inhibiting malignant lung cells from dissemination (Fig. 5).

**Figure 6:**
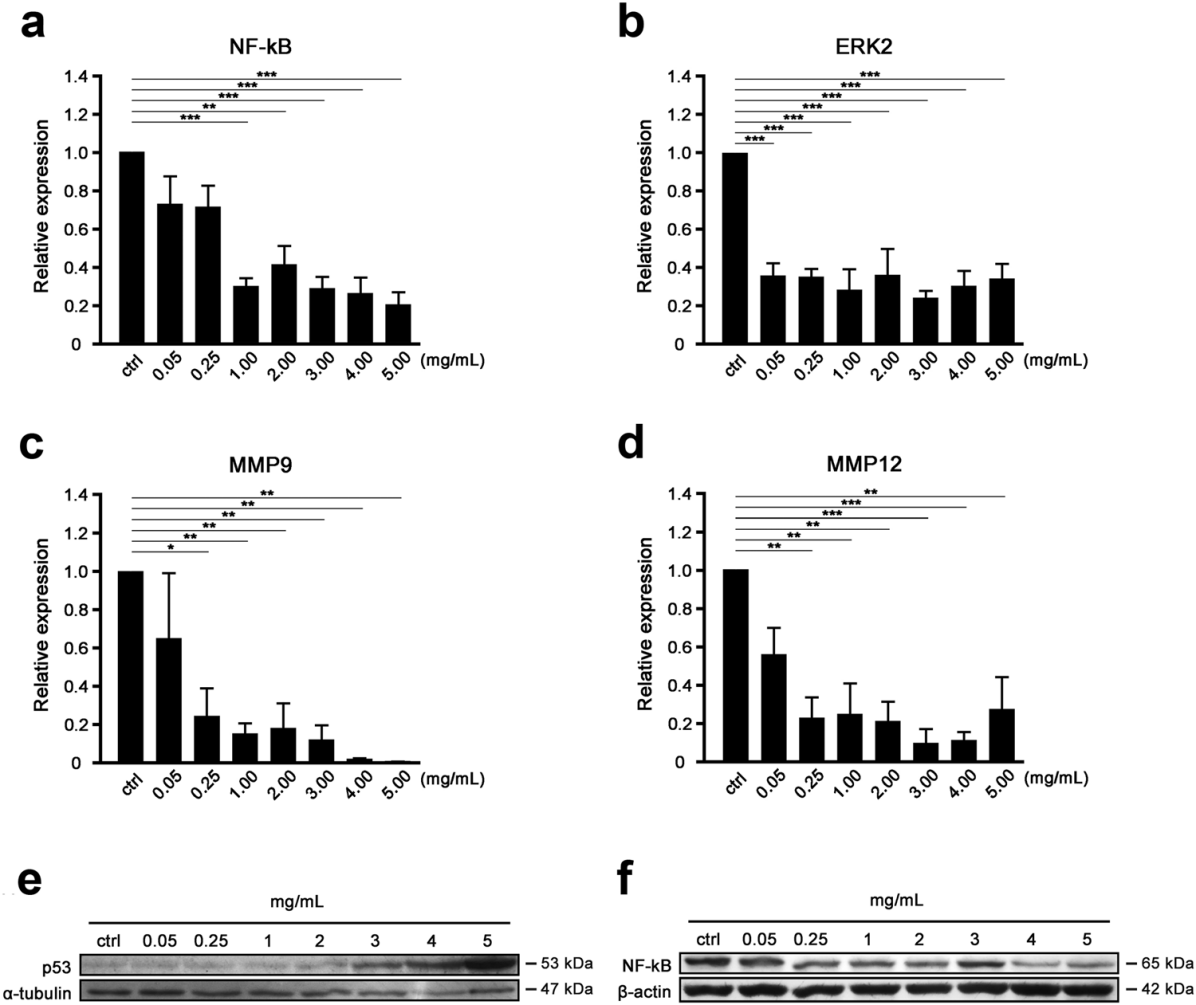
Gene and protein expression analysis of A549 cells treated with SaM. (**a**–**d**) Quantitative real-time PCR analysis of the mRNA expression levels of NFκB, ERK2, MMP9, and MMP12. Statistical differences between the control and the treated groups were determined by a one-way ANOVA followed by the Dunnett’s post-hoc test when results of the ANOVA were significant: *P < 0.05, **P < 0.01, ***P < 0.001 vs. control. Western blot analysis of A549 cells before and after SaM treatment. Cropped blots were probed with anti-p53 (**e**) and NF κB antibodies (**f**). In the figure are reported the cropped gels/blots obtained by each protein evaluation. All gels were run in the same experimental conditions (see material and methods for details). Full-length blots are presented in Supplementary Figure S5.

To delineate potential intracellular modulator(s) and molecular pathway (s) that were responsible for the observed cytotoxic property, effect of SaM treatment on gene and protein expression were studied in A549 cells. Extensive data strongly support the idea that biological disturbances are required for the onset, progression, and chemoresistance of cancer through activation of critical cellular transcriptional factors such as nuclear fatoctor-κB (NF-κB)^12^. This ubiquitous transcription factor has been shown to be constitutively active in many tumor cell lines, including ones with lung origin^13–16^. The up-regulation and activation of NF-κB has been implicated in the control of proliferation and apoptosis in many types of cancer cells^17,18^ and the anti-apoptotic function of NF-κB poses a major obstacle to effective radiation therapy and chemotherapy^12,19^. Therefore, NF-κB and its signaling pathways are considered important targets for therapeutic intervention in managing many types of cancer^20,21^. Studies have demonstrated that the deregulation of NF-κB pathway in NSCLC could play a crucial role in the development and progression of the disease^22–24^. Tumor samples obtained from lung cancer patients showed high levels of NF-κB in NSCLC^7^. Moreover, study showed that inhibition of this proliferation/survival modulator chemosensitized NSCLC^25^. Our quantitative real-time PCR and western blot analyses of NF-κB in treated A549 cells showed that the gene/protein expression of this proliferation/survival modulator was significantly reduced in A549 cells exposed to very low dose of SaM (Fig. 6a,f). SaM exhibiting inhibitory effect on NF-κB expression in A549 cells signifies the use of SaM at targeting NF-κB in combination with either traditional or novel chemotherapeutic agents in lung cancer treatment.

The mRNA expression of another key molecular modulator involved in tumor growth, *ERK2*, was also reduced in A549 cells exposed to very low dose of SaM (≥0.05 mg/mL), as shown in Fig. 6b. Despite *KRAS* being one of the earliest known oncogenetic drivers NSCLC^26,27^, effective targeting of this molecule remains a therapeutic challenge since direct RAS inhibition (e.g. with salirasib) was unsuccessful^27^. Novel approaches are currently attempting to inhibit downstream molecules in the RAS/RAF/MEK/ERK pathways. Selumetinib (AZD6244; ARRY-142866) a MEK1/MEK2 inhibitor showed a progression-free survival advantage when combined with docetaxel in a recent phase II trial in advanced *KRAS*-mutant NSCLC^28,29^. Here we reported that SaM (≥0.05 mg/mL) was effectively at reducing the expression of *ERK2* and our finding provides a new lead on finding alternative and combined chemotherapeutic treatment in managing NSCLC by targeting the RAS pathway. Interestingly, molecules involved metastasis, *MMP9* and *MMP12*, were also down-regulated in mRNA expression with low dose SaM treatment (≥0.25 mg/mL; shown in Fig. 6c,d), in support of our finding that SaM attenuates malignant cell migration.

Finally, our western analysis of the protein level of the key death factor/antitumor molecule p53 demonstrated that the protein expression was elevated in A549 cells treated with ≥2 mg/mL SaM in a dose-dependent manner (Fig. 6e). *p53* gene mutations are among the most frequent molecular events in carcinogenesis, found in approximately 60% of cases of NSCLC and 90% of small cell lung cancer^30,31^. The high prevalence of *p53* mutations in the early course of tumorigenesis suggests that loss of normal *p53* function is an important step in NSCLC oncogenesis and tumor progression^32–34^. Thus, targeted inhibition/down-regulation of NF-κB and activation/up-regulation of p53 by using SaM would be a promising mean and approach to treat NSCLC. Interestingly, the very first link between p53 and necrosis was only recently reported and signified the direct relationship between reactive oxygen species-induced necrosis and p53^4^. Using H_2_O_2_ treatment of cultured cells to model necrosis in ischemic tissues, p53 was also found to be required for necrotic cell death activation^1^. In line with these observations, our data provide compelling evidence that p53 and H_2_O_2_ have novel and pivotal roles as a modulator of necrosis. It is well documented that p53 suppresses tumor growth by interfering cell cycle. Nevertheless, our cell cycle analysis showed that exposure to SaM did not affect cell cycle kinetics and progression delay of cells through the cell cycle phases was not noted (Supplementary Figure S2).

### ***In Vivo*** Toxicological Assessment (hematological and histological analyses)

In the present study, we reported that SaM was cytotoxic to human lung adenocarcinomic cells *in vitro*. Therefore, it became our immediate interest to determine whether SaM have *in vivo* antitumor activity against diseased lung cells. To ensure that the treatment of SaM does not induce vital organ damage and acute immunotoxicity (i.e. immunosupression) in our study animals, histological and hematological analyses were carried out. As shown in Supplementary Table S1, body and vital organ weights of animals administered with low (2 mg/animal /day) and high doses (6 mg/animal/day) of SaM were comparable to that of control and water-fed animals. Lung, kidney, and liver tissues were all normal in treated animals, confirming that SaM did not induce overall organ toxicity (Supplementary Figure S3). Hematological analysis showed that only the red blood cell counts and the hemoglobin content were elevated in animals fed with SaM (Supplementary Figure S4a,g); however, these counts were within normal and healthy range (Charles River Laboratories International Inc. hematology report/http://www.criver.com/files/pdfs/rms/cd1/rm_rm_r_cd1_mouse_clinical_pathology_data.aspx^35^), suggesting that SaM might be immunoenhancing. Analysis of peripheral blood composition (T cell, B cell, granulocyte, and macrophage) showed that production of cells in four major lymphopoetic and myelopoetic lineages were not adversely suppressed in experimental animals, as shown in Supplementary Figure S4c–f, demonstrating that SaM did not induce acute immunotoxicity. Finally, the production of full lineages of blood cells relies on the hematopoietic stem cells, which dictate blood cell formation and ultimately sustain a normal immune system. Treatment of SaM did not alter the production of hematopoietic stem cells (LSK) (Supplementary Figure S4h), reconfirmed that SaM was not immunosuppressive and exhibited no adverse effect on hematopoietic stem cells in hosts.

### *In Vivo* Assessment of Antitumor Property

**Figure 7:**
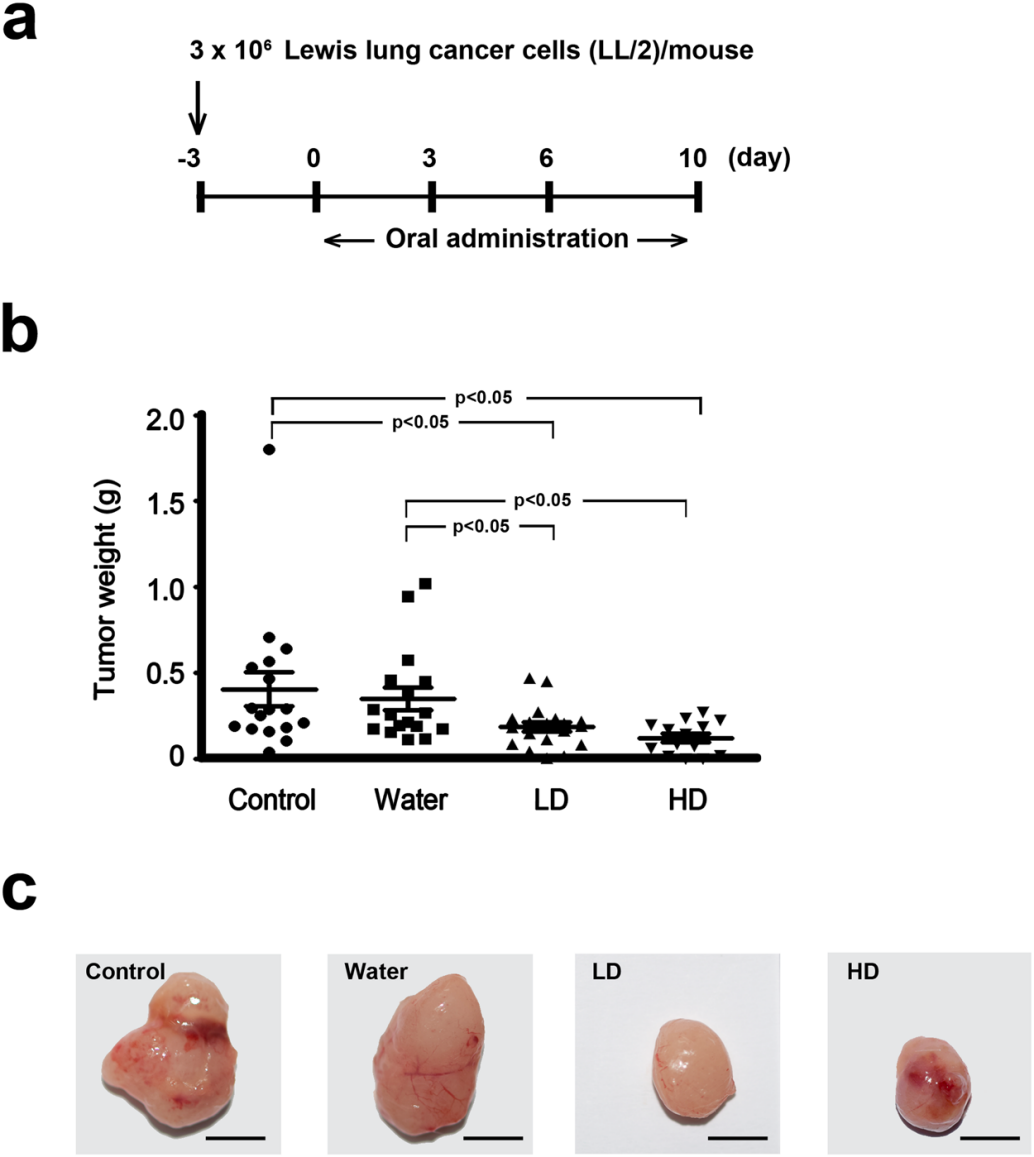
*In vivo* assessment of antitumor property of SaM using LL/2-bearing animal model. (**a**) Treatment protocol of tumor-bearing mice. Mice were fed orally with water, low- or high dose of SaM for total 10 days. (**b**) Tumor weights of control, water-fed, and SaM treated ICR mice. LD = low dose (2 mg/mouse); HD = high dose (6 mg/mouse). (**c**) Representative photographs of inoculated LL/2 tumors from the control and treated animals. Scale bar = 1 cm. The data are expressed as individual tumor weight and horizontal bar represent are the mean values. Statistical differences between the control and the treated groups were determined with a Mann-Whitney Rank Sum Test and results were considered significant when p < 0.05 vs. control.

**Figure 8:**
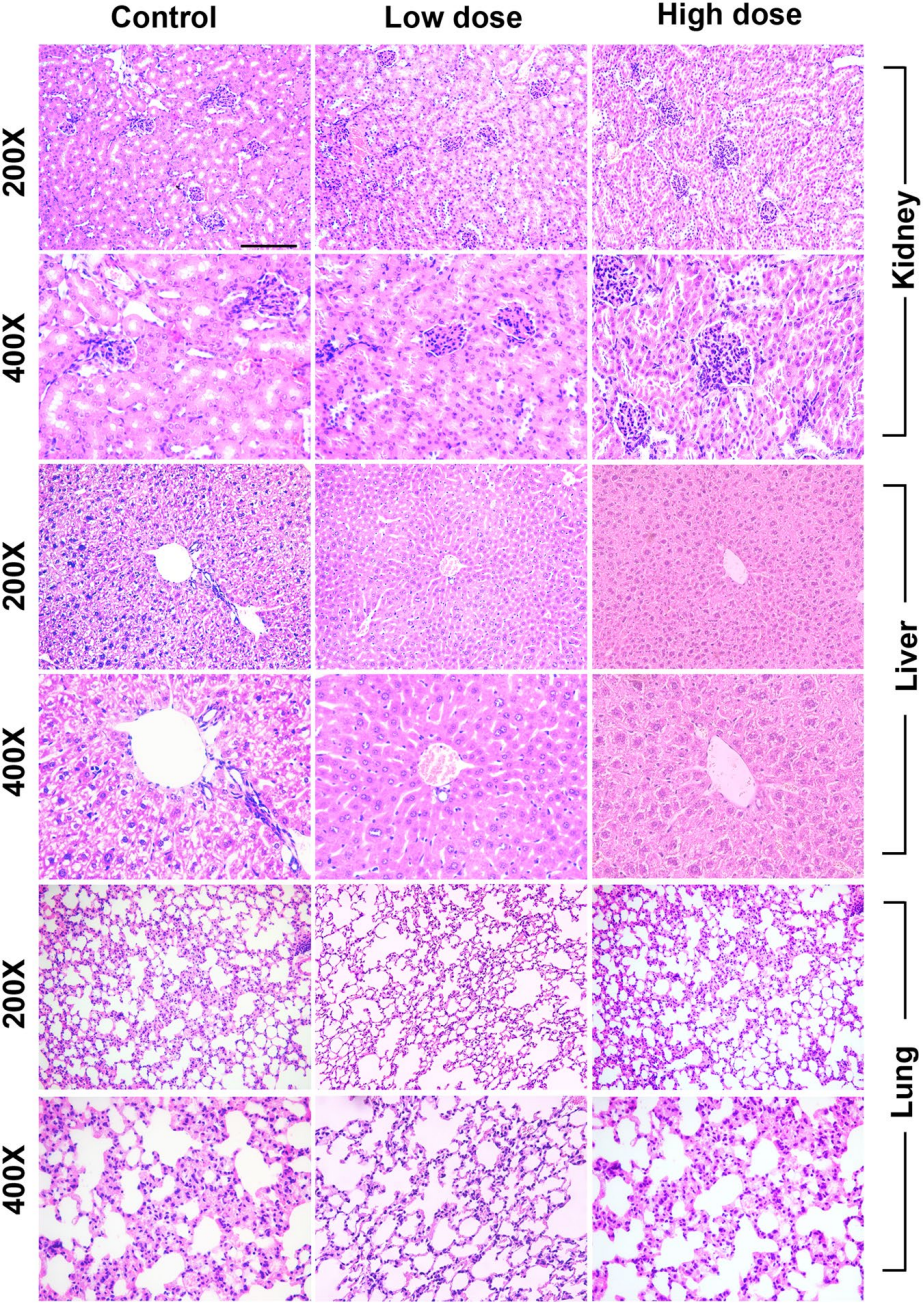
*In vivo* toxicological assessment of LL/2-bearing ICR mice treated with SaM. H&E staining of major organs (kidney, liver, and lung). Micrographs were taken at 200× and 400× magnifications. Scale bar = 100 um.

**Figure 9:**
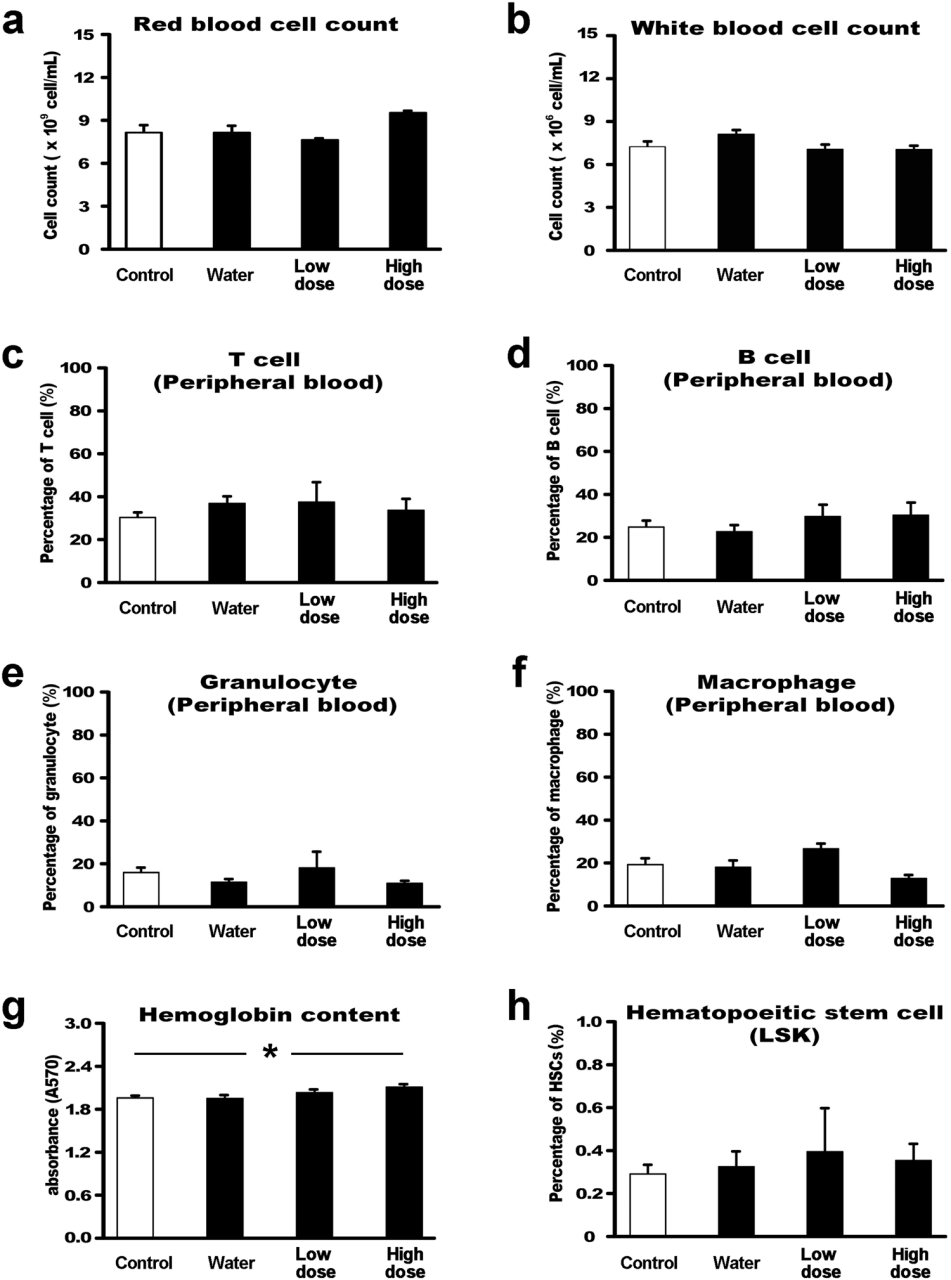
Hematological analysis of LL/2-bearing ICR mice treated with SaM. Low dose = 2 mg/mouse; High dose = 6 mg/mouse. (**a**) Red blood cell and (**b**) white blood cell counts of control, water-fed, and SaM treated mice. Data are expressed as average total cell number ± SEM. (**c**–**f**) Leukocyte analysis: T cell, B cell, granulocytes, and macrophage cell counts. Data are expressed as average percentage ± SEM. The average percentage was calculated by dividing the number of T cells, B cells, granulocytes, or macrophage cells by total number of leukocytes. (**g**) Hemoglobin content of peripheral blood. Data are expressed as absorbency at wavelength of 570 nm ± SEM. (**h**) Hematopoietic stem cell (LSK) analysis. Average percentage of hematopoietic stem cell (±SEM) was calculated by dividing the number of LSK cells by total viable bone marrow cells. Statistical differences between the control and the treated groups were determined with a one-way ANOVA followed by the Dunnett’s post-hoc test when results of the ANOVA were significant: *p < 0.05 vs. control.

To evaluate whether SaM has *in vivo* antitumor activity against diseased lung cells, animals bearing murine Lewis lung cancer LL/2 were administered with SaM (low dose: 2 mg/animal/day; high dose: 6 mg/animal/day) and inhibitory effect of SaM treatment on the bearing tumors was examined (Fig. 7a). Remarkably, administration of SaM significantly reduced the average tumor weights (control: 0.41 ± 0.10 g; low dose: 0.19 ± 0.03 g; high dose: 0.13 ± 0.03 g) in experimental animals (Fig. 7b). Animals fed with water only did not show significant tumor weight reduction (0.35 ± 0.07 g). There was no significant difference in tumor weights of animals fed with low and high doses of SaM. Representative photographs of inoculated tumors collected from the control and experimental animals are shown in Fig. 7c. Body weights and weights of major vital organs of both control and experimental animals were not significant different (Table 2). Histological structures of lung, kidney and liver tissues were also normal in treated animals, reconfirming that SaM was not toxic to animals (Fig. 8). Hematological analysis of all animals showed that all blood parameters were with in healthy range and animals in high dose group had higher hemoglobin content in red blood cells (Fig. 9). As shown in our *in vitro* study, the cytotoxic property of SaM was necrosis-based. One of the main concerns regarding this type of cell death was the “off-target” effect by inducing inflammation in normal cell/tissues of the hosts. Data from our hematological analysis presented here showed that antitumoral SaM is not pro-inflammation (i.e. all tested animals showed normal white blood cell count and macrophage percentage in peripheral blood) and should be considered safe to use. In conclusion, SaM is effective at inhibiting lung cancer cell growth *in vivo* without undesired toxicity and side-effects.

**Table 2:** Mean organ and body weights of ICR mice bearing murine Lewis lung cancer LL/2.

Groups	Organ and body (g)					
	Liver	Kidney	Spleen	Pancreas	Digestive system	Body weight
Control	2.12 ± 0.09	0.29 ± 0.03	0.17 ± 0.04	0.25 ± 0.04	3.72 ± 0.21	36.67 ± 2.05
Water	2.23 ± 0.12	0.29 ± 0.03	0.15 ± 0.01	0.24 ± 0.04	3.98 ± 0.31	34.27 ± 1.65
Low dose	1.52 ± 0.08	0.29 ± 0.01	0.10 ± 0.01	0.14 ± 0.00	4.64 ± 0.37	35.06 ± 1.69
High dose	1.98 ± 0.22	0.34 ± 0.03	0.18 ± 0.04	0.22 ± 0.05	4.02 ± 0.36	36.27 ± 2.76

Organ and body weights of control, water-fed, and SaM treated ICR mice. Low dose = 2 mg/mouse; High dose = 6 mg/mouse. Data are expressed as mean organ/body weights ± SEM. Statistical differences between the control and the treated groups were determined by a one-way ANOVA followed by the Dunnett’s post-hoc test when results of the ANOVA were significant: *p < 0.05 vs. control.

## Conclusion

We discovered that *S*. *mukorossi* leaf and stem aqueous extract, named SaM, was rich in polysaccharide. SaM was more potent at reducing adenocarcinomic lung cells than fractions of SaM (fraction I or II). SaM was cytotoxic *in vitro* by inducing oxidative stress and necrotic cell death, in addition to attenuating cell invasiveness. Moreover, SaM was antitumoral *in vivo* by reducing lung tumor weight without inducing organ damage or any undesired side effects such as immunosuppression or inflammation. This work, to our knowledge, is the first study documents the antitumor bioactivity of aqueous extract rich in polysaccharides from *S*. *mukorossi* against diseased lung cells. SaM has advantages of low toxicity, oral bioavailability, and findings from this work provide promising leads on the development of an alternative carbohydrate-based therapeutic regimen for lung cancer treatment.

## Materials and Methods

### Chemical and Reagents

Cell culture media were purchased from Hyclone (Logan, UT). NF-κB (SC-372), p53 (SC-126), α-tubulin (SC-5546), rabbit-anti-mouse HRP conjugated (SC-358923) antibodies were purchased from Santa Cruz Biotechnology (Santa Cruz, CA). β-actin antibody (MAB-1051) was purchased from Millipore. Goat anti-rabbit HRP (A-10547) conjugated antibody was purchased from Invitrogen (Waltham, MS).

### Preparation of Aqueous Plant Crude Extracts SaM and Fraction I/II

Fresh whole plants were collected in Zhi-Shan Yan (25°5′38″N, 121°30′57″E) on June, 2013 and the geographic information was provided by voucher specimen (153786) from the Herbarium of National Taiwan University and voucher specimen (69363) from the PH Herbarium, Academy of Natural Sciences, Philadelphia. Specimens were authenticated by taxonomists at the Department of Life Science, Chinese Culture University, Taiwan, and stored at −80 °C prior to plant crude extraction. Plant materials (stem and leaf) were cleaned with deionized water to remove adulterant first and air-dried at room temperature. Leaves and stems were then pulverized separately into fine powders. Aqueous extract SaM was made by incubating equal weight of stem and leaf powders in nanopure water in a ratio of 1:1:3 (w:w:v; stem powder weight in gram: leaf powder weight in gram: water volumn in milliliter) overnight at 4 °C without agitation. SaM was then filtered with Whatman^TM^ no. 5 filters (2.5 μM) to remove plant debrits and the dry constitutents were obtained by freez-drying or centrifugal concentration. To prepare fraction I/II, extract SaM was centrifuged for 10 min at 3000 rpm and the supernatant was then concentrated by freeze-dried and the yield was ~7.71%. The dry constituents of SaM was reconstituted in deionized water in a ratio of 2.7:10 (w:v) and fractionated by HPLC with OHPak SB803 HQ (20 × 300 mm) column to collect fractions I (<6 kDa) and fraction II (>6 kDa). Fraction I and II were freeze-dried and reconstituted in cell culture media for *in vitro* cytotoxicity tests.

### High-Performance Anion-Exchange Chromatography (HPAEC) Analysis of the Carbohydrate Composition of SaM

For carbohydrate composition analysis, SaM was lyophilized and subjected to acid hydrolysis. One milligram of lyophilized aqueous extract was hydrolyzed with 1.95 N trifluoroacetic acid (TFA) at 80 °C for 6 h. The mixture was cooled, evaporated and then resuspended in nanopure water. Monosaccharides were separated on an HPAEC system (Dionex BioLC) equipped with a gradient pump, a pulsed amperometric detector (PAD-II) using a gold working electrode, and an anion-exchange column (Carbopac PA-10, 4.6 × 250 mm). Hydrolysate was applied using an autosampler (AS3500, SpectraSYSTEM^®^) via a microinjection valve with a 200 μl sample loop. The analysis of monosaccharides was carried out at an isocratic NaOH concentration of 18 mM at ambient temperature. Identification and quantification of monosaccharides were made in comparison with standards. Data were collected and integrated on a PRIME DAK system (HPLC Technology, Ltd. UK).

### Animals

Animal maintenance and usage were approved by Chinese Culture University institutional animal care and use committee (CCU-IACUC-10314). ICR male mice were purchased from The Experimental Animal Center of National Taiwan University (Taipei, Taiwan). All mice were housed with 14:10 light and dark cycle and fed water and rodent chow ad libitum. All animals were handled and received humane care in compliance with the principles of laboratory animal care. All methods were performed in accordance with the approved guidelines and regulations.

### Cell Culture

Human adenocarcinomic alveolar basal epithelial cell line A549 and murine Lewis lung cancer cell line LL/2 were purchased from Taiwan Bioresource Collection and Research Center (Taipei, Taiwan). A549 and LL/2 were passaged in Dulbecco’s modified eagle’s medium (DMEM) supplemented with 10% (v/v) FBS. To perform *in vitro* assays and gene/protein expression analysis of A549 cells treated with SaM, cultures were initiated and the formation of monolayer cells was visually confirmed 24 hours later. Cultures were then administered with SaM at the final concentrations as specified for 72 hours. All control groups were not exposed to SaM.

### Cell Proliferation Assay

To assess cell proliferation, cultured cells were harvested with 0.05% trypsin-EDTA and detached cells from each culture were enumerated using hemocytometer by trypan blue exclusion assay. Cell morphology was grossly examined and recorded using inverted phase contrast microscope. Cell proliferation was expressed as total number of live cell.

### Cell Viability Assay

Cell viability was assessed by resazurin based method (PrestoBlue® reagent, Invitrogen). In brief, sample cells were incubated with PrestoBlue® for 4 hours under standard culture condition according to manufacturer’s suggestions. The amount of dye generated was quantified by measuring the absorbency at 570 nm. Viability was defined as the ratio of absorbency of treated cells to untreated cells, and data were expressed in mean percentage ± SEM.

### Oxidative Stress Analysis

Oxidative stress was assessed by quantifying the intracellular production of reactive oxygen species H_2_O_2_, using dichloro-dihydro-fluorescein diacetat (DCFH-DA). Detection of fluorescence in cells was interpreted as evidence of H_2_O_2_ production and oxidative stress. Samples were stained with 5 μm/mL DCFH-DA at 37 °C for 60 minutes and intracellular fluorescence was examined microscopically. The intracellular generation of H_2_O_2_ was defined as the ratio of DCF fluorescence positive cells to total cells, and the mean number of oxidative stressed cells was expressed in percentage ± SEM.

### Cell Death Analysis

Apoptosis and necrosis were assessed flow cytometrically by Annexin V binding assay (BD Pharmingen™, New Jersey). In brief, cells were collected and washed in HBSS, supplemented with 10 mM HEPES and 2% FBS (HBSS-H/2%). Samples were then stained with Annexin V-FITC for 30 minutes at room temperature. Annexin V positive cells (PI-positive or -negative) were defined as apoptotic cells and PI positive only cells were defined as necrotic cells. All data were collected using Becton Dickinson FACSCalibur flow cytometer (BD, New Jersey) and analyzed using FlowJo software. Data were expressed in percentage ± SEM.

### DNA Fragmentation Analysis

To perform DNA fragmentation analysis, genomic DNA was isolated from >5 × 10^5^ cultured cells by cell lysis and alcohol precipitation. The purified DNA was electrophoresed analyzed using a 1.5% agarose gel with ethidium bromide staining.

### Scratch Migration Assay

Scratch migration assay was performed as described previously^35,36^. In brief, cells were seeded at 50–60% confluency for 24 hours before scratch migration assay was performed. A scratch of the cell monolayer was created and cultures were replaced with the culture medium with SaM. The rate of cell migration was determined 24 and 48 hours later. Migration was defined as the relative of average migrating distance of treated cells to untreated cells. Average migrating distance was determined by measuring 20 readings of distance for each sample and data were expressed as rate of migration (%).

### RT-qPCR Analysis

Total RNA was isolated using TRIzol reagent (Invitrogen, Carlsbad CA) according to the manufacturer’s suggestions. RNA was reverse transcribed using Superscript III and random primers (Invitrogen) and the resulting cDNA was stored in aliquots at −80 °C until use. Real-time PCR with SYBR green (Life Technologies ABI; Calsbad CA) detection was carried to measure the relative expression of NF-κB (forward primer: ACCCTGACCTTGCCTATTTG, reverse primer: AGCTCTTTTTCCCGATCTCC), ERK2 (forward primer: AGATTCCAGCCAGGATACAGAT, reverse primer: AGACAGGACCAGGGGTCAA), MMP9 (forward primer: GAACCAATCTCACCGACAGG, reverse primer: GCCACCCGAGTGTAACCATA), MMP12 (forward primer: GGAATCCTAGCCCATGCTTT, reverse primer: CGTGAACAGCAGTGAGGAAC). GAPDH (forward primer: AGCCACATCGCTCAGACAC, reverse primer: GCCCAATACGACCAAATCC) was used as a housekeeping gene for relative expression analysis. Dissociation analysis was performed at the end of each run to confirm the specificity of the reaction.

### SDS-PAGE and Western Blotting

Proteins were separated by SDS-PAGE and were blotted onto nitrocellulose. Blots were then probed with appropriate antibodies according to manufacturer’s suggestion. In brief, protein blots were blocked with 1–5% BSA in Tris-buffered saline (TBT: 147 mM NaCl, 20 mM Tris-base and 0.1–1% Tween-20, pH 7.6) for 1 h and incubated with primary antibody with appropriated concentration in TBT with constant agitation overnight at room temperature. Blots were then probed with appropriated horseradish-conjugated secondary antibody in TBT for 1 h at room temperature. Bands of interest were detected by enhanced chemiluminescence assay and band images were captured by exposing to Konica MG-SR Plus X-ray films. Band intensity was analyzed by Image J. Gels/blots used in figures are in compliance with the digital image and integrity policies.

### Hematological Analysis

Mice (8–12 wks weeks) with similar body weight were divided into four groups: group 1 was served as a control group without any treatment; group 2 received water once a day for 10 days; group 3 received total 2 mg SaM (low dose) in water once a day for 10 days; group 4 received 6 mg SaM (high dose) in water once a day for 10 days. To perform red and white blood cell counts, peripheral blood was collected from the heart and treated with acid citrate dextrose-based anti-coagulant solution. For hemoglobin content assessment, erythrocytes from the peripheral blood were lysed and the released hemoglobin was quantified spectrophotometrically by measuring the absorbency at 570 nm. Mouse hematopoietic stem cells were referred here as LSK (Lin^-^Sca-1^+^cKit^+^) cells and defined as lineage negative (CD3e, CD11b, CD45R, Ly-G/C6, TER-119, NK-1.1), Sca-1(Ly-6A/E) and c-Kit (CD117) positive^37^. To detect LSK, bone morrow collected from femur and tibia were stained with Sca-1-specific PE antibody, c-Kit-specific APC antibody, and biotinylated lineage-specific antibodies (CD3e, CD11b, CD45R, Ly-6G/C, TER-119, NK-1.1) followed by streptavidin-conjugated Alexa 488. Prior to flow cytometric analysis, all samples were stained with 1 μg/mL PI and the percentage of LSK cells in bone marrow was analyzed. All data were collected using Becton Dickinson FACSCalibur flow cytometer (BD, New Jersey) and analyzed using FlowJo software.

### Histological Analysis

For histological analysis, paraffin embedded tissue sections were stained with hemotoxylin and eosin (H&E). In brief, liver, kidney, and lung were removed and washed with cold PBS prior to fixation in 4% of paraformaldehyde. Tissues were then dehydrated using a series of graded ethanol followed by paraffin infiltration. Infiltrated tissues were then embedded into paraffin wax blocks and 5 μm sections were cut for HE staining. For lung histological analysis, the presence of gas exchange areas such as alveolar sacs was examined. For liver histological analysis, the presence of portal triad elements consisted of hepatic artery branch, hepatic vein, and bile duct was examined. The general morphology of liver parenchyma and hepatocytes were evaluated. For kidney histological analysis, the presence of glomeruli with surrounding Bowman’s capsule and convoluted tubes in the cortex was examined.

### *In vivo* Antitumor Analysis

Male ICR mice (8–12 wks) with similar body weight were inoculated by subcutaneous injection of 3 × 10^6^ murine Lewis lung cancer cell line LL/2 cells in 200 uL PBS into the right flank of the mouse. Three days after LL/2 inoculation, mice were divided into four groups: group 1 was served as a control group without any treatment; group 2 received water once a day for 10 days; group 3 received total 2 mg SaM (low dose) in water once a day for 10 days; group 4 received 6 mg SaM (high dose) in water once a day for 10 days. Mice were then sacrificed and tumors were removed, photographed, and weighted. Hematological and histological analyses were carried out as described in above sections (see section 4.14 and 4.15).

### Combination (CI) Index Calculation

CI index of fraction I and II were calculated using software CompuSyn (ComboSyn Inc, New Jersey). CI index quantitatively depicted the synergism (CI < 1), additive effect (CI = 1), or antagonism (CI > 1) as described previously^38^.

### Statistical Analysis

Data were expressed as mean ± SEM and analyzed statistically using a one-way analysis of variance (ANOVA) followed by Dunnet’s post-hoc test. All data were collected from ≥3 independent experiments. In both the one-way ANOVA and the Dunnet’s test, results were considered significant when p < 0.05. For *in vivo* antitumor experiments, data were analyzed using Mann-Whitney Rank Sum Test and results were considered significant when p < 0.05.

### Data availability statement

The datasets generated during and/or analyzed during the current study are available from the corresponding author on reasonable request.

## Electronic supplementary material

Supplementary Information
