# Supplementary material for: Aqueous extract of Sapindus mukorossi induced cell death of A549 cells and exhibited antitumor property in vivo
Source: Sci Rep. 2018 Mar 19;8:4831. doi: 10.1038/s41598-018-23096-w (PMC5859273; doi:10.1038/s41598-018-23096-w)
Supplement: Supplementary file 1 — Supplementary Information [file 41598_2018_23096_MOESM1_ESM.pdf]

TITLE

Aqueous extract of *Sapindus mukorossi* induced cell death of A549 cells and exhibited antitumor property *in vivo*

Authors

Min Liu[1,2‡], Yen-Lin Chen[1], Yao-Haur Kuo[3,4,5], Mei-Kuang Lu[3], Liao Chia Ching[6]

Affiliated Institutes

1. Department of Life Science, Chinese Culture University, Taipei, Republic of China
2. Graduate Institute of Biotechnology, Chinese Culture University, Taipei, Republic of China
3. Ministry of Health and Welfare, National Research Institute of Chinese Medicine, Taipei, Republic of China
4. Graduate Institute of Integrated Medicine, China Medial University, Taichung, Republic of China
5. Department of Horticulture and Biotechnology, College of Agriculture, Chinese Culture University, Taipei, Republic of China
6. Department of Biology, Chinese Culture University, Taipei, Republic of China

[‡] **Corresponding Author**

Min Liu, Ph.D.

Mailing address: Department of Life Science, Chinese Culture University, Taipei, Republic of China

Phone: +886228610511

Fax: +886228623724

E-mail address: lm@faculty.pccu.edu.tw

Supplementary Materials and Method

Nuclear magnetic resonance (NMR) and Evaporative light scattering detection (ELSD) analysis

S. mukorossi aqueous crude extract SaM was analyzed by NMR and the spectra were measured using a Unity Plus 400 MHz NRM (Brucker BioSpin, Germany) with DMSo-d_6 as the D-solvent. Spectrum was analyzed using Brucker Topspin 2.1 software. ELSD analysis was carried out in conjunction with high performance liquid chromatography (HPLC). OHPak SB804 (8.0 x 300 mm) column was used and analysis condition was set at 40°C with a flow rate of 1.0 mL/min. In brief, aqueous samples was lymphosized and was reconsttituted with deionized water (1:30). Sample solution was then centrifuged and debrides was removed with #1 filter paper. Filtration using 0.22 μm syringe filters was performed prior to ESLD analysis.

Cell Cycle Analysis

To perform cell cycle analysis, flow cytometry-based method was employed. In brief, both control cells and treated cells were harvested by trypsinization and washed with PBS to make single cell suspension. Samples were then fixed with cold methanol over night at 4°C. On the next day, cells were washed and resuspended in PI staining solution (10 ug/mL), supplemented with 100 ug/mL of DNAse-free RNAse A, made in PBS. Samples were kept from light and incubated at 37°C for 60 minutes. DNA contents of all stained samples were analyzed using Becton Dickinson FACSCalibur flow cytometer (BD, New Jersey). ModFit LT software was used for post analysis for the percentage of cells in G_1/G_0, S, and G_2/M phases. Data were presented as percentage of cells in G_1/G_0, S, and G_2/M phases.

Supplementary Figure

A

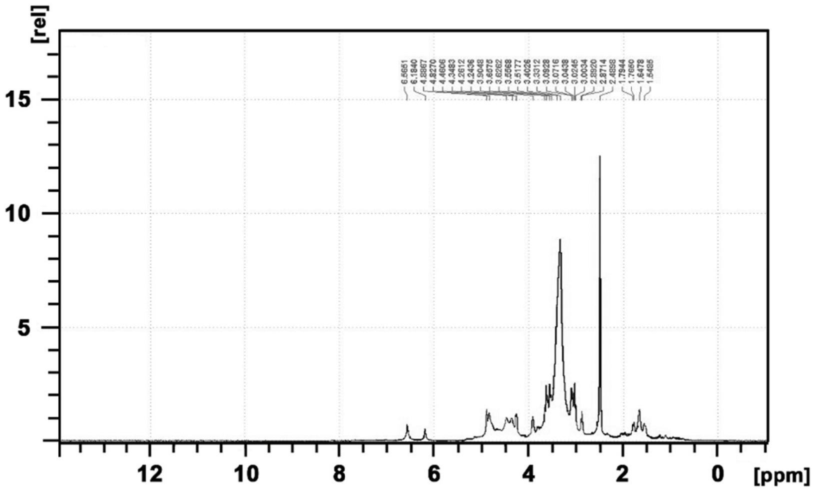

B

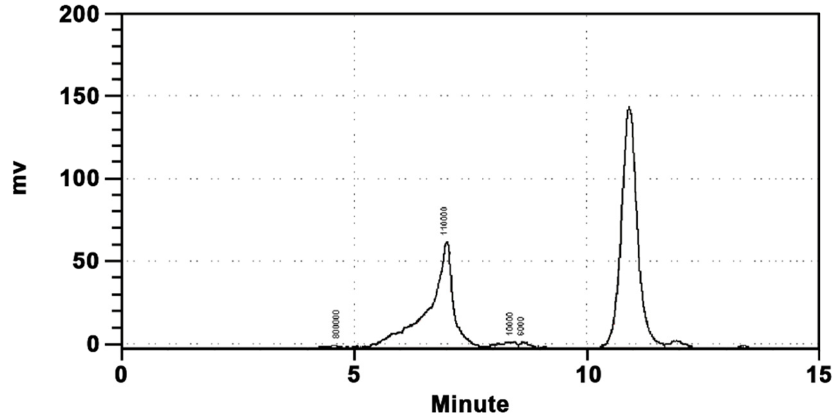

Supplementary Figure S1

Chemical analyses of SaM. (A) ^{1}H-NMR spectrum of SaM. (B) Evaporative light scattering detector (ELSD) chromatograph of SaM. Two major peaks (fraction I and II) were detected. Fraction I (> 3000 Da) and II (>89000 Da) were collected for further study.

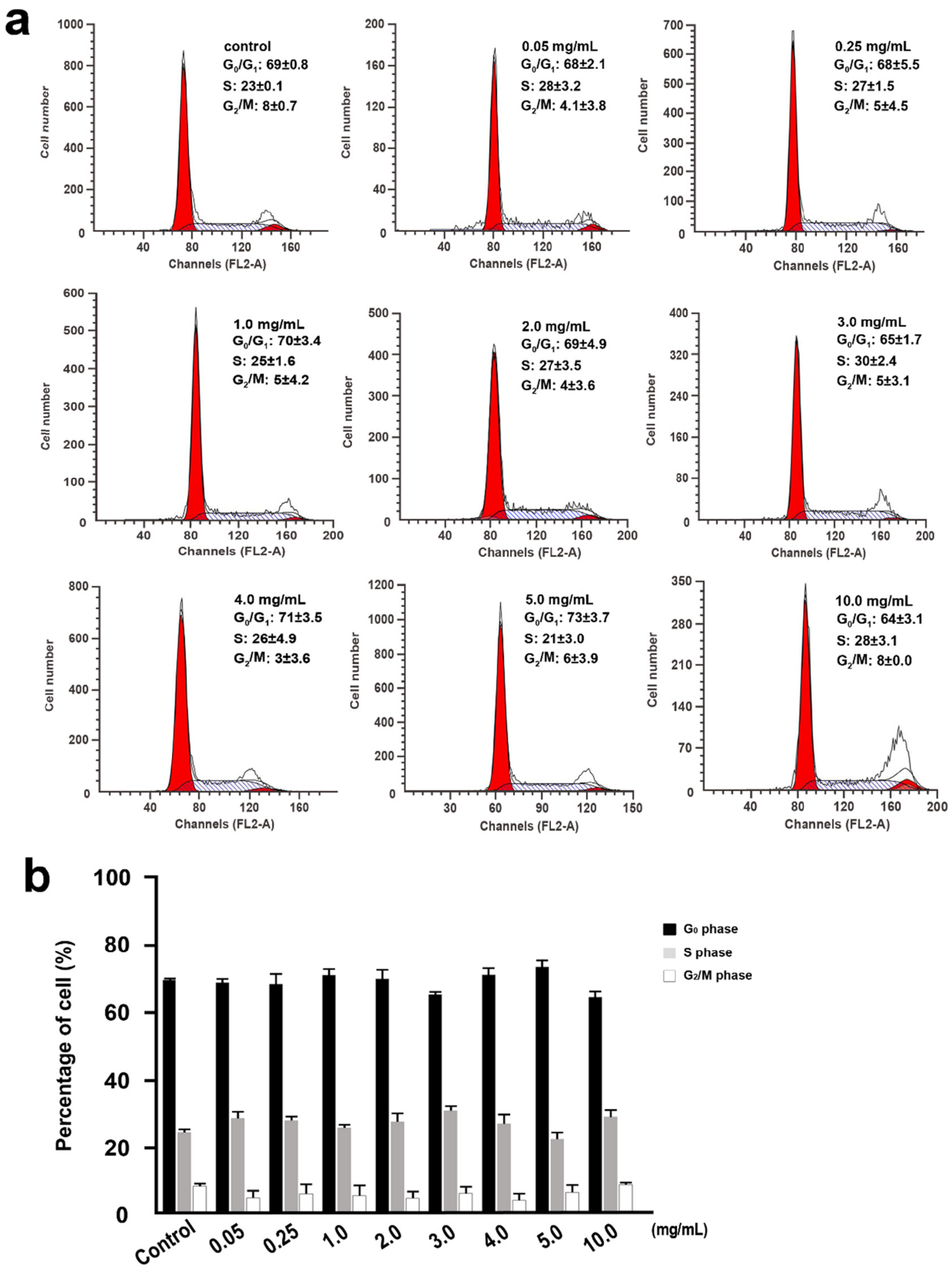

Supplementary Figure S2

Flow cytometric cell cycle analysis of A549 cells treated with SaM. The experimental groups were administered with SaM (0.05 -10 mg/mL) for 48 hours. Control groups were not exposed to the crude extract. (a) The number of cells in G_0/G_1, S, G_2/M and the percentage (± SEM) of cells in these cell cycle phases were shown. The representative flow cytometric patterns of control and treated groups are shown. (b) The bar graphs showed the

percentage (± SEM) of cells in G_0/G_1 (black bar), S (grey bar), G_2/M (white bar) phases of control and treated groups. The experiments were conducted in triplicate. Statistical differences between the control and the treated cell in G_0/G_1, S, G_2/M were determined with a one-way ANOVA followed by the Dunnett's post-hoc test when results of the ANOVA were significant: $^{*}p<0.05$ vs. control.

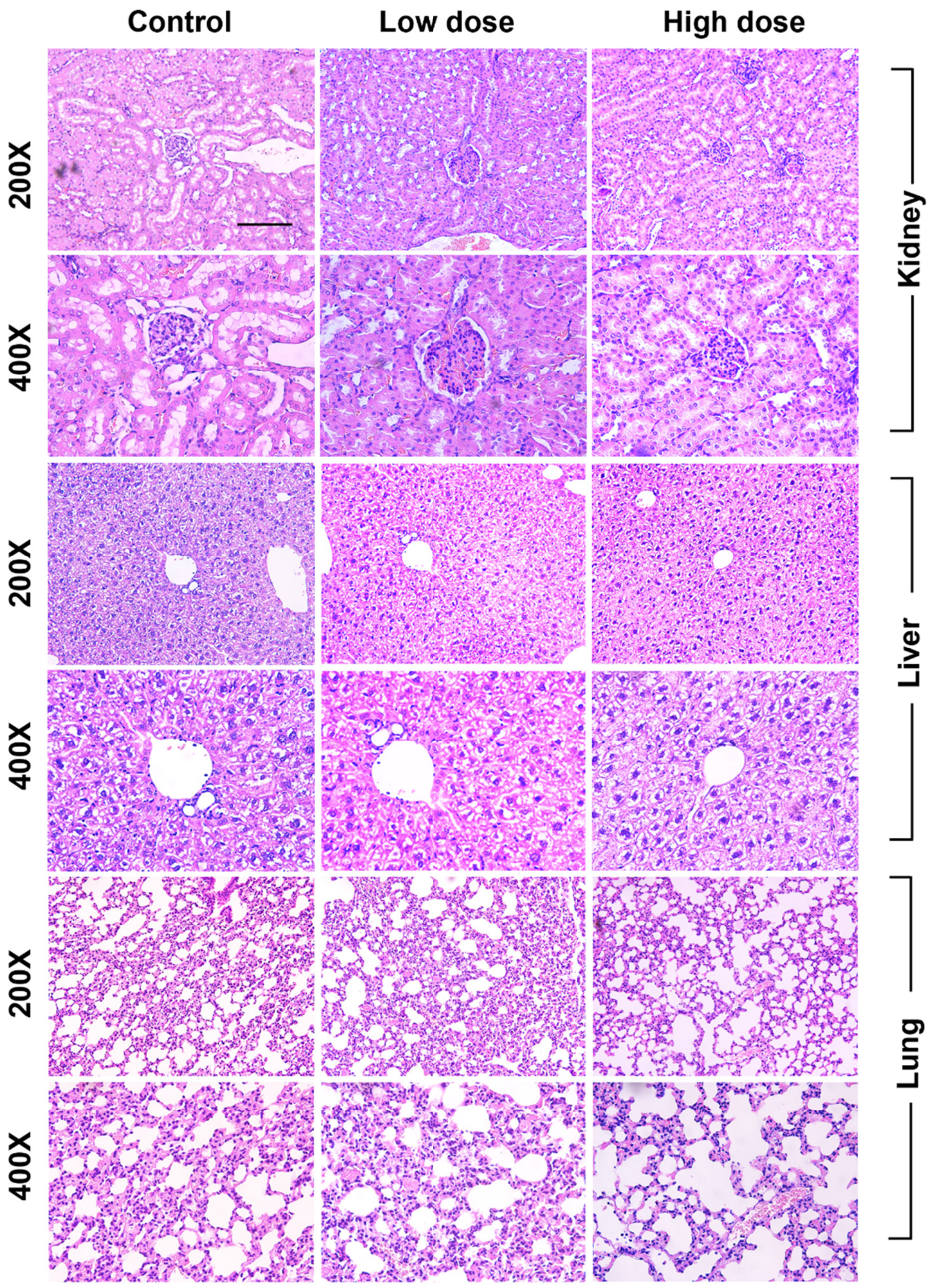

Supplementary Figure S3

In vivo toxicological assessment of SaM. H&E staining of major organs of (kidney, liver and lung). Micrographs were taken at 200x and 400x magnifications. Scale bar = 100 um.

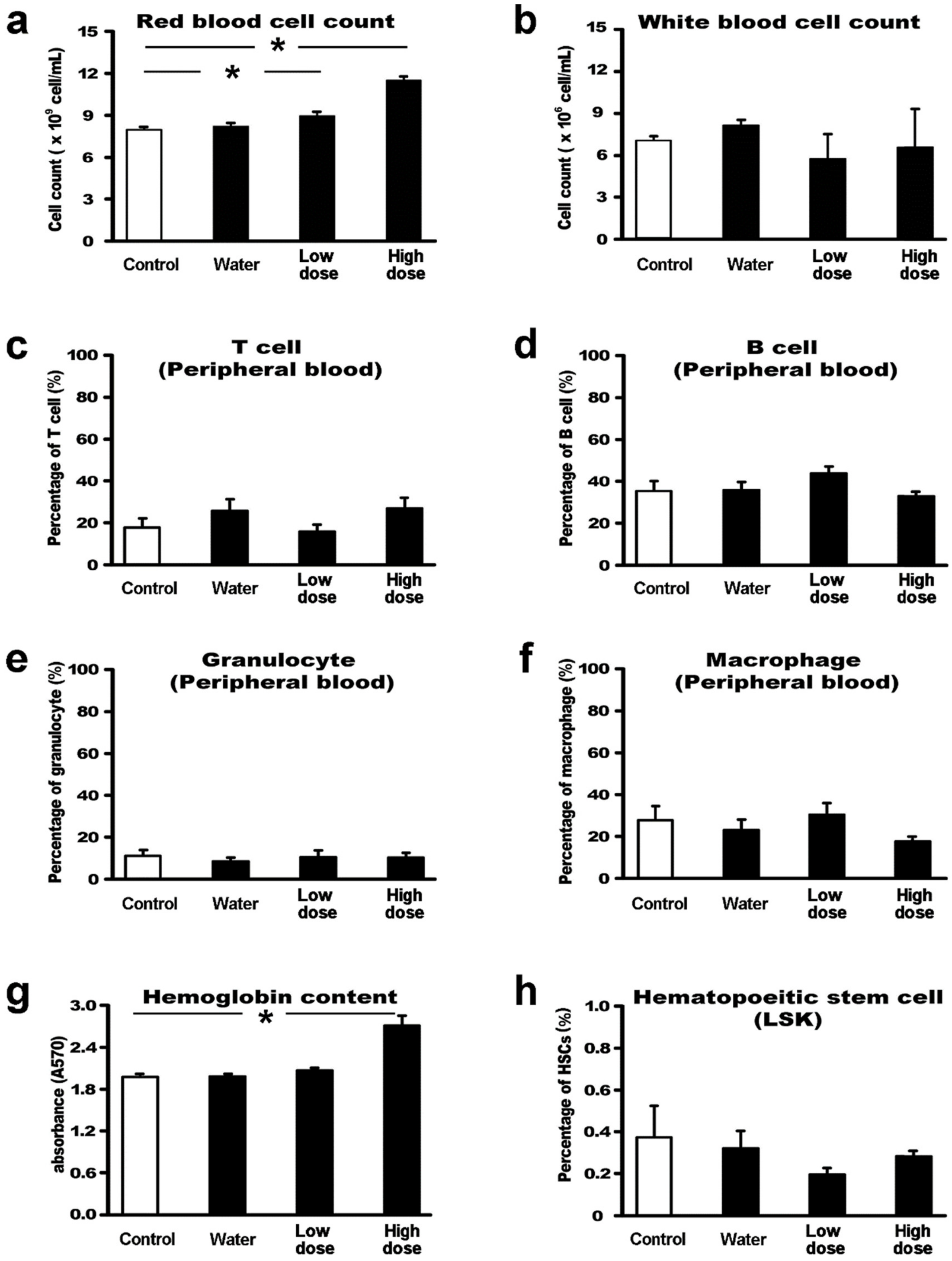

Supplementary Figure S4

Hematological analysis of SaM treated ICR mice. Low dose = 2mg/mouse; High dose = 6mg/mouse. (a) Red blood cell and (b) white blood cell counts of control, water-fed, and SaM treated mice (low dose and high dose). Data are expressed as average total cell number ± SEM. (c – f) Leukocyte analysis: T

cell, B cell, granulocyte, and macrophage cell percentages. Data are expressed as average percentage of each cell lineage ± SEM. The average percentage was calculated by dividing the number of T cells, B cells, granulocytes, or macrophage cells by the total number of leukocytes. (g) Hemoglobin content of peripheral blood. Data are expressed as absorbency at wavelength of 570 nm ± SEM. (h) Hematopoietic stem cell (LSK) analysis. Average percentage of hematopoietic stem cell ± SEM was calculated by dividing the number of LSK cells by total viable bone marrow cells. Data were obtained from > 3 independent experiments. Statistical differences between the control and the treated groups were determined with a one-way ANOVA followed by the Dunnett's post-hoc test when results of the ANOVA were significant: *$p<0.05$ vs. control.

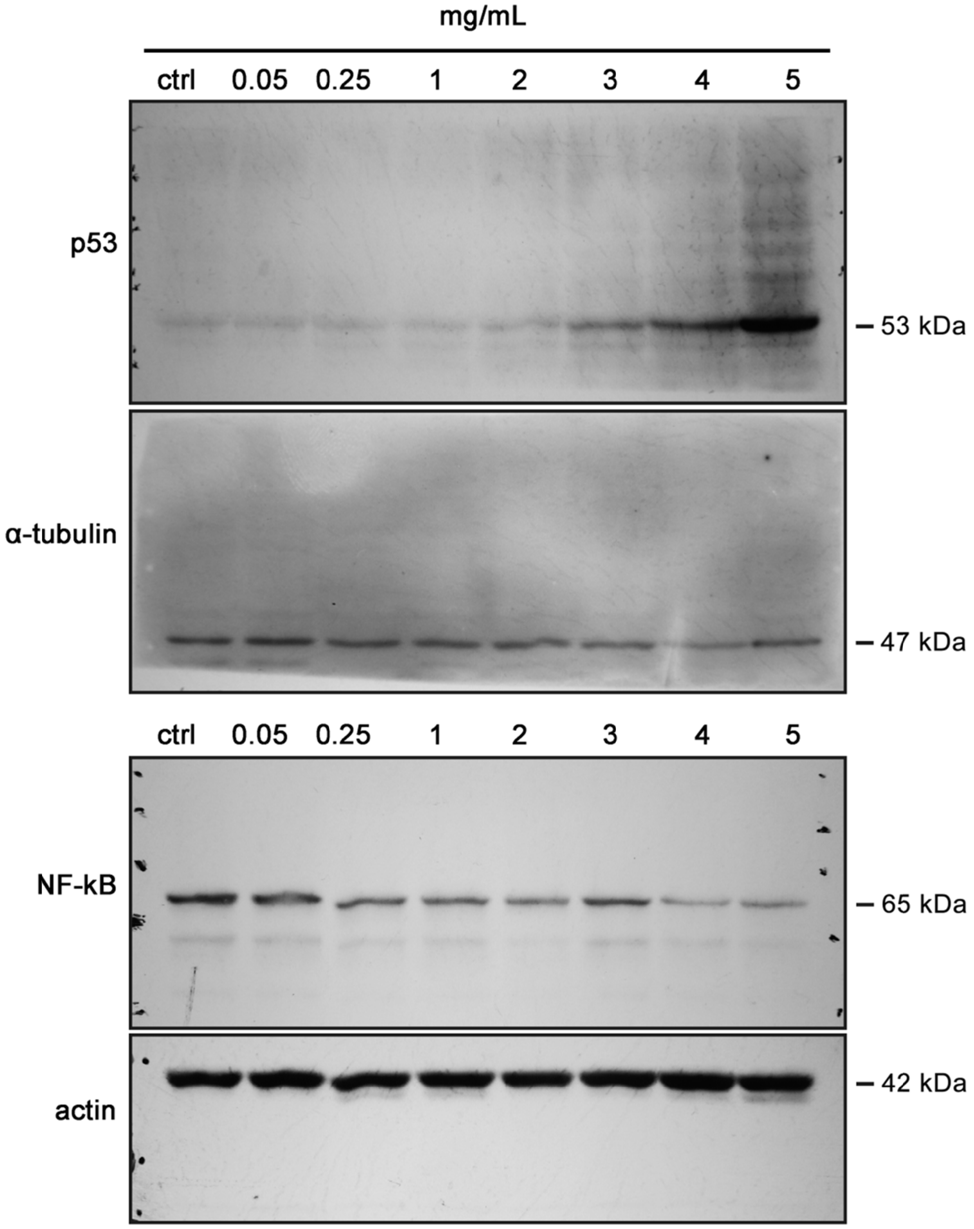

Supplementary Figure S5

Full length blots of Figure 6e and 6f. Specific protein evaluation and the corresponding control protein bands were obtained from the same gel/blot.

Supplementary Table S1. Mean organ and body weights of ICR mice

	Organ and body (g)					
**Groups**	**Liver**	**Kidney**	**Spleen**	**Pancreas**	**Digestive system**	**Body weight**
**Control**	1.91 ± 0.24	0.29 ± 0.04	0.15 ± 0.03	0.20 ± 0.03	4.95 ± 0.47	34.53 ± 0.53
**Water**	1.80 ± 0.17	0.31 ± 0.04	0.12 ± 0.01	0.22 ± 0.01	4.56 ± 0.19	33.77 ± 0.53
**Low dose**	1.86 ± 0.09	0.30 ± 0.04	0.13 ± 0.00	0.23 ± 0.02	5.24 ± 0.30	34.70 ± 0.52
**High dose**	2.38 ± 0.16	0.27 ± 0.04	0.11 ± 0.00	0.20 ± 0.01	4.67 ± 0.22	36.38 ± 0.65

Organ and body weights of control, water-fed, and SaM treated ICR mice. Low dose = 2mg/mouse; High dose = 6mg/mouse. Data are expressed as mean organ/body weights ± SEM. Statistical differences between the control and the treated groups were determined by a one-way ANOVA followed by the Dunnett's post-hoc test when results of the ANOVA were significant: *$p<0.05$ vs. control.
